# Current News and Opinion

**Published:** 1888-08

**Authors:** 


### Current News and Opinion.

### CORRESPONDENCE.

*Editor Independent Practitioner:—*

In your July number I noticed a desire for squibs, in consequence of which I submit the following: In the discussions upon copper amalgams, that have appeared in dental journals of late, as well as the discussion in the Mass. and Conn. Valley Union meeting recently, I noticed that some prominent members of the profession have claimed absolute non-union between the copper and other amalgams. I have placed copper amalgam in the mouth, and topped off and contoured with a quick-setting amalgam, and obtained the most satisfactory results. The copper makes an excellent lining for sensitive and deep cavities, and when topped with a quick-setting amalgam we have a most perfect preservative filling. I have experimented with three different kinds of amalgams, or alloys, in union with copper amalgam made by myself according to Bloxam, in glass tubes and otherwise, and found absolute union. Copper amalgam can be made antiseptic by the addition of a few drops of dilute-hydrochloric acid, which has considerable affinity both for the copper and the mercury, thereby hastening amalgamation, and the chlorine uniting with the mercury to form bichloride ($HgCl_2$), or $H_2$ $SO_4$ concentrated could be used; add common salt in excess and wash and have a good antiseptic amalgam. It would have to be used intelligently.

H. M. CLIFFORD, D. M. D.
Boston.

---

*Editor Independent Practitioner.*

DEAR SIR:—I wish to report, through the columns of your journal, a very unusual, if not anomalous, case. On June 11th I was called by John Bell, M. D., physician in charge, to see a child, born the evening before, whose jaws could not be opened. As it had been a breech presentation, and considerable force had been necessary, the doctor feared that some displacement might have occurred.

We found, upon careful examination, the parts in position, but the following conditions existing: Quite a marked protrusion of the anterior part of the sup. maxillary bones, not excessive, but making it about as long as the upper lip, the upper edge of the inf. maxillary resting against, and slightly back of, the upper, a marked recession of the chin; the whole state of affairs giving the mouth and lips somewhat the appearance of being puckered, as in whistling. A small spatula could be passed between the jaws (the mucus membrane covered them naturally) entirely around on the right side, but on the left only a little beyond the corner of the mouth; the jaws appeared to be joined.

By the exercise of considerable force we could separate the jaws in front about a line, but could perceive no motion at the joints, and we came to the conclusion that all we gained was a slight springing of the parts.

Our diagnosis was that there must be bony union between the sup. and inf. maxillary bones. The child lived just a week, and after its death we obtained the consent of the parents to a partial dissection of the parts, when we found the bones coalesced, from about opposite the mental foramen on the left side entirely back to where the joint should have been, but of which there was no trace.

The appearance was that of one bone, the periosteum extending across, and with no groove or depression to mark the line of union between the bones. The other parts seemed natural, the child full grown and healthy.

Dr. Bell says the knee of the child rested directly under the chin at birth, and the father informs me that they lost another child in convulsions when the mother was about three months advanced in her pregnancy. She also suffered very much during the time with her teeth, and would, as he expressed it, "continually grate them together in her sleep." We very much desired to preserve the case for more thorough examination and deposit in some museum, but it was impossible, without resort to such means as are almost out of the question here.

H. C. ROCKWELL, D. D. S.

BENTON HARBOR, Mich., July 16, 1888.

NOTE.—These communications are just what are wanted. Who will be the next to send in something?—EDITOR.

---

### BIBLIOGRAPHICAL.

THE SUPERIOR INCISORS AND CANINE TEETH OF SHEEP. By FLORENCE MAYO. With two plates. Reprinted from Bulletin of the Museum of Comparative Zoology at Harvard College. Vol. XIII., No. 9. June, 1888.

The work reported in this memoir was done under the direction of Prof. E. L. Mark, and reflects credit upon the laboratory. The writer draws the following conclusions from the study of a long line of cross serial sections made through the superior maxilla from the milian line backwards, and including the primolars (1): "that in the embryo sheep at a certain stage of development the dental lamina exists throughout the canine and incisor regions of the upper jaw. Its anterior portion, which is *last* to develop and the first to abort, does not attain so prominent a condition as its lateral portions. After advancing in development for a time, it retrogrades, and finally disappears (2); that in the canine the dental lamina gives rise to an enamel germ which never reaches a stage of functional activity, for neither are its central cells transformed into a stellate reticulum, nor do those of the malpighian layer ever produce enamel, and in later stages both disappear.

"In this region there is no trace of a dentine germ. The fact of the existence in sheep of rudiments of such organs as usually result in the formation of teeth is of interest because it is one of those peculiar structures for which it is difficult to account without the aid of the theory of natural selection. From the observations here recorded one readily sees that the disappearance of the superior incisors and canines is progressive. In the region of the incisors the evidences even of the beginnings of tooth development have almost disappeared, the region of the first incisor being the least differentiated portion of the tract, while the canine region is represented by a moderately large but functionless enamel sac. Since in some ruminants destitute of incisors small rudimentary canine teeth are found on the upper jaw of the adult animal, it is a fair inference that the teeth are being lost from before backward, and that the canine teeth, the last to disappear from the sheep, are in such cases undergoing degeneration, although not wholly functionless.

"If it is admitted that the history of the development of the individual reproduces, at least in part, the history of the ancestors of that individual, and that the changes in development take place in the same order as in the ancestors, then we have reason for believing that the progenitors of the ruminants possessed incisors and canine teeth on the upper jaw; that these teeth becoming, perhaps by a change in environment, no longer necessary for obtaining food, have gradually ceased to develop, and that the disappearance of the teeth has been a progressive process, beginning with the middle incisors and gradually involving the teeth farther back."

CAMBRIDGE, September, 1887.

---

### ABOUT RAILWAYS.

*Scribner's Magazine* is publishing a series of very interesting railway articles, and from them has been derived the following questions and answers which convey a great amount of useful information:—

Q. How many miles of railway in the United States? A. 150,600 miles; about half the mileage of the world.

Q. How much have they cost? A. $9,000,000,000.

Q. How many people are employed by them? A. More than 1,000,000.

Q. What is the fastest time made by a train? A. 92 miles in 93 minutes; one mile being made in 46 seconds, on the Phil. and Reading R. R.

Q. What is the cost of a high-class eight-wheel passenger locomotive? A. About $8,500.

Q. What is the longest mileage operated by a single system? A. Atchison, Topeka & Santa Fe system, about 8,000 miles.

Q. What is the cost of a palace sleeping car? A. About $15,000, or $17,000 if "vestibuled."

Q. What is the longest railway bridge-span in the United States? A. Cantilever span in Poughkeepsie bridge, 548 feet.

Q. What is the highest railroad bridge in the United States? A. Kinzua Viaduct, on the Erie Road, 305 feet high.

Q. Who built the first locomotive in the United States? A. Peter Cooper.

Q. What road carries the largest number of passengers? A. Manhattan elevated railroad, New York; 525,000 a day, or 191,625,000 yearly.

Q. What is the average daily earning of an American locomotive? A. About $100.

Q. What is the longest American railway tunnel? A. Hoosac tunnel, on the Fitchburg railway, (4¾ miles.)

Q. What is the average cost of constructing a mile of railroad? A. At the present time about $30,000.

Q. What is the highest railroad in the United States? A. Denver & Rio Grande; Marshall Pass, 10,852 feet.

Q. What are the chances of fatal accident in railway travel? A. One killed in ten million. Statistics show more are killed by falling out of windows than in railway accidents.

Q. What line of railway extends furthest east and west? A. Canadian Pacific railway, running from Quebec to the Pacific Ocean.

Q. How long does a steel rail last, with average wear? A. About eighteen years.

Q. What road carries the largest number of commuters? A. Illinois Central, 4,828,128, in 1887.

Q. What is the fastest time made between Jersey City and San Francisco? A. 3 days, 7 hours, 39 minutes and 16 seconds. Special Theatrical Train, June, 1886.

---

### EYE INFLAMMATION DEPENDENT UPON LESIONS OF DENTAL BRANCHES OF THE FIFTH NERVE.

Dr. F. W. Marlow reports three cases illustrating the dependence of eye inflammation upon irritative lesions of the dental branches of the fifth nerve.

The following is a summary of his paper:—

1. The inflammation was most marked at the margin of the cornea. 2. The conjunctival affection was limited to congestion and slight swelling, there being no discharge of matter. It would thus seem probable that the conjunctival symptoms were simply secondary to the corneal. 3. The corneal affection was quite superficial, but extended over a considerable area. In the first case it much resembled that seen in inherited syphilis. In the two last cases there was a tendency to loss of epithelium. Thus it differed from the ordinary phlyctenular type of keratitis, which tends to be more definitely localized in one or more spots, and to be accompanied by well marked ulceration, and the development of blood-vessels on the cornea.

4 In all the cases there were variations in the condition from time to time, the symptoms never entirely disappearing (with the exception of those of the left eye in one case), in spite of the long-continued use of the usual local and general treatment.

5. When the dental irritation was removed, the eye-symptoms disappeared rapidly and completely in each case, and so far without relapse.

There can be little doubt, he says, that the removal of the dental irritation and the disappearance of the eye-symptoms stood to one another in the relation of cause and effect, and he thinks it may fairly be said that the dental irritation aggravated and prolonged the eye-inflammation, if it did not, as indeed seems very probable, constitute the original exciting cause of the trouble. If we admit, he concludes, that the condition of the teeth influenced the eye-trouble in any way in these cases, it becomes evident that a dental lesion too slight, or at any rate not of a nature to call attention by symptoms to itself, may yet be sufficient to exert such an influence.—*Medical and Surgical Reporter.*

### TRANSPLANTATION OF BONE.

H. M. Sherman briefly reviews this important and interesting subject, and reports two apparently successful cases.

He regards the following cases of importance: That a perfectly healthy bed of granulations must first be secured—preferably by means of iodoform gauze packings. The grafts should be taken from the bone of some young and rapidly-growing animal, and from as near an epiphysis as possible, that the utmost vital action may be secured. They should consist of both bone and periosteum, and perhaps even cartilage of ossification may be of advantage. Their maximum size should not exceed one-third of an inch in length and one-quarter of an inch in breadth. Grafting has been successfully done with human, kid, and dog bones. These are probably the best sources. The grafts should be so planted that they will have free vascular connections on all sides, for such was their condition before transplantation. This may be accomplished by incisions into granulation beds. The grafts must be held down by *moderate* pressure, so that they shall not be forced from their location by newly-forming granulations.

Strict, but not too powerful, antisepsis should prevail, so that the discharges may be kept at a minimum and sweet.

The dressings should not be changed more often than is necessary to secure this desideratum; hence, should be large and very absorptive —*Pacific Medical and Surgical Journal, January,* 1888

---

### PECULIAR METHOD OF THE TRANSMISSION OF SYPHILIS.

Tepliachine writes of a peculiar Russian custom. Among the peasantry many explain almost every affection of the eyes as due to the presence of foreign bodies between the lids, where such bodies may be retained. In order to extract them the tongue of some person is introduced between the lids and into the conjunctival sacs where the supposed foreign body is lodged The procedure is very widespread, and, as may be readily understood, syphilis may in this way very easily be transmitted. The author describes a veritable epidemic of syphilis in the government of Wiatka. Within two months he had in his hospital eight cases of syphilis of the lids (with mucous patches, also, of the anus and genitalia). The relatives of the patients themselves stated that the syphilitic virus had been thus inoculated by a woman of the village that everybody thought a female physician. The author examined this woman, and found upon her all the symptoms of syphilis. He also discovered in the neighboring hamlets sixty-eight syphilitic cases, amounting to thirteen per cent. of all the population, one-half of whom, or thirty-four, were inoculated by the same woman —*Wratch, April.*

---

### CONCENTRATED SOLUTION OF BORIC ACID.

According to the Paris correspondent of the *British Medical Journal*, June 2, 1888, M. A. Cabanès in the *J. de Méd. de Paris*, April 15, has discovered a new way of dissolving a large quantity of boric acid in distilled water. It is known that it has not hitherto been possible to dissolve more than 4 or 4.50 parts of this antiseptic in 100 parts of water, which is not strong enough completely to destroy all micrococci. The new solution is as follows: boric acid, 120 parts; calcined magnesia, 10 parts; aquæ distill., 750 parts. It is possible that biborate of magnesium is formed; at any rate, there remains in solution a considerable excess of boric acid.

### NAPHTHOL $\beta$ AS AN ANTISEPTIC.

In a communication recently made by Professor Bouchard to the Académie des Sciences, he states that naphthol $\beta$ is an excellent antiseptic for external use. But what is more important, it would appear to be the most efficient agent for intestinal antisepsis, particularly on account of its slight solubility, which prevents its absorption, and allows it to remain a long time in the intestine without poisonous effects. A daily variable dose of 1 gramme of naphthol per kilogramme of weight of the animal may be administered without danger.—*Brit. Med. Journ.*, June 2, 1888.

---

THE following officers were elected at the eighteenth annual meeting of the New Jersey State Dental Society, to serve for the ensuing year:

*President*—H. A. Hull, of New Brunswick.

*Vice-President*—S. C. G. Watkins, of Montclair.

*Treasurer*—George C. Brown, of Elizabeth.

*Secretary*—C. A. Meeker, of Newark.

*Executive Committee*—George E. Adams, South Orange; Oscar Adelberg, Elizabeth; B. F. Luckey, Paterson; C. W. F. Holbrook, Newark.

*Board of Examiners*—J. Hayhurst, Lambertville; A. R. Eaton, Elizabeth; Frederick C. Barlow, Jersey City; James G. Palmer, New Brunswick; Frederick A. Levy, Orange.

---

THE Missouri State Dental Association, at its twenty-fourth annual meeting, elected the following officers to serve for the ensuing year:

*President*—B. Q. Stevens, Hannibal.

*First Vice-President*—T. W. Reed, Mancon.

*Second Vice-President*—W. E. Tucker, Butler.

*Recording Secretary*—John G. Harper, St. Louis.

*Corresponding Secretary*—William Conrad, St. Louis.

*Treasurer*—James A. Price, Weston.

The twenty-fifth annual meeting will be held at Pertle Springs, Warrensburgh, beginning on the first Tuesday after the Fourth of July, 1889.

JOHN G. HARPER, Rec. Sec.

---

### AMERICAN DENTAL ASSOCIATION.

The American Dental Association will hold its twenty-eighth annual meeting at Louisville, Ky., commencing Tuesday, August 28, 1888.

GEO. H. CUSHING,
Recording Secretary.

### SPECIAL RATES TO THE JOINT MEETING AT LOUISVILLE.

One and one-third fare rates have been secured over lines belonging to the—

Southern Passenger Association,
Central Traffic Association,
Texas Traffic Association, and
The Trunk Line.

The Chesapeake & Ohio Railroad will sell round-trip tickets for a single fare from Washington, Richmond, Charlottsville, and all points on their line.

Tickets on sale August 24, 25, and 26—good till September 10.

In order to secure the benefit of the reduction in rate, a certificate must be obtained from the agent at the time of purchase. These, when signed by the Secretary of the Dental Association, will entitle the bearer to a return ticket at one-third rate. Tickets are non-transferable, and good for return only over the same route traversed in going.

---

### NATIONAL ASSOCIATION OF DENTAL FACULTIES.

The National Association of Dental Faculties will meet in a fifth annual session at the Gault House, in the city of Louisville, Ky., at 9 A. M., on Monday, August 27, 1888.

In order that this meeting may be dispatched before the Southern and American Dental Associations get to work, it is hoped that the entire membership may be represented promptly at the hour indicated.

A. O. Hunt, President,
Iowa City, Iowa.

Junius E. Cravens, Secretary,
Indianapolis, Ind.

---

### THE NATIONAL ASSOCIATION OF DENTAL EXAMINERS.

The next meeting of the National Association of Dental Examiners will be held in Louisville, Ky., on Monday evening, August 27th, at 8 o'clock, and at other times during the week, between the sessions of the American and Southern Dental Associations. It is important to have every State Board represented.

Fred A. Levy, D. D. S., Secretary.

---

### PEROXIDE OF HYDROGEN.

Maklakoff recommends peroxide of hydrogen, not only as a therapeutic agent, but also for diagnostic purposes. This is because of its peculiarity of penetrating the interstices of tissues during the period of its decomposition. It thus becomes a powerful antiseptic remedy, and in setting free large quantities of gas it renders visible the spaces, furrows, etc., that one would not see, and whose existence would otherwise be unsuspected.—*Med. Russe, January*, 1887.

